# Life‐Threatening Prolonged ICANS Following Tarlatamab Treatment in Extensive‐Stage Small Cell Lung Cancer With Brain Metastases: A Case Report

**DOI:** 10.1002/rcr2.70617

**Published:** 2026-05-14

**Authors:** Hiroyuki Arai, Shinji Sasada, Hiroki Kazahari, Yuya Homma, Kyohei Kaburaki

**Affiliations:** ^1^ Department of Respiratory Medicine The Fraternity Memorial Hospital Tokyo Japan; ^2^ Division of Medical Oncology, Department of Internal Medicine Teikyo University School of Medicine Tokyo Japan

**Keywords:** ICANS, small cell lung cancer, tarlatamab, toxicity

## Abstract

We report the case of a 75‐year‐old woman with extensive‐stage small cell lung cancer (ES‐SCLC) who developed severe, prolonged immune effector cell‐associated neurotoxicity syndrome (ICANS) following tarlatamab. She had previously received first‐line carboplatin, etoposide, and durvalumab, followed by second‐line amrubicin. As third‐line therapy, she received tarlatamab and developed Grade 4 ICANS within 18 h. Recurrent seizures required emergent intubation and mechanical ventilation for 6 days. Although her immune effector cell‐associated encephalopathy (ICE) score transiently improved after extubation, it subsequently deteriorated to 0–1, leaving her minimally responsive. Contrast‐enhanced brain MRI revealed multiple previously unrecognized brain metastases. The absence of allergic predisposition or prior neurologic symptoms suggested that untreated intracranial disease, rather than host factors, contributed to the unusually severe and prolonged ICANS. This case highlights that occult or untreated brain metastases may increase susceptibility to life‐threatening ICANS during tarlatamab therapy.

## Graphical abstract

A patient with extensive‐stage small cell lung cancer developed life‐threatening, prolonged ICANS shortly after tarlatamab treatment. Brain MRI revealed previously unrecognized multiple brain metastases, suggesting a possible link between occult intracranial disease and severe neurotoxicity.

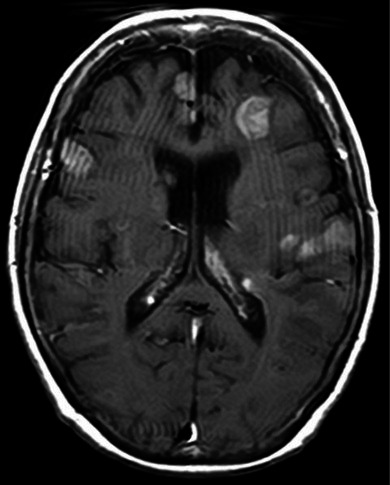

## 1 Introduction

Tarlatamab is a bispecific antibody that binds CD3 on T cells and DLL3 on tumour cells, inducing T‐cell–mediated cytotoxicity in small cell lung cancer (SCLC). A characteristic adverse event is immune effector cell‐associated neurotoxicity syndrome (ICANS), which can be severe and may present with altered consciousness and seizures [1]. We describe a patient with extensive‐stage SCLC (ES‐SCLC) who developed life‐threatening, prolonged Grade 4 ICANS after the first dose of tarlatamab, in whom multiple brain metastases were detected only after neurotoxicity developed.

## 2 Case Report

A 75‐year‐old woman with ES‐SCLC and liver metastases (cT3N3M1c, stage IVB) was admitted to initiate third‐line tarlatamab after prior systemic therapies. She had initially been diagnosed with SCLC during evaluation for abdominal pain and severe hyponatremia (serum sodium 104 mmol/L). After sodium correction with hypertonic saline and tolvaptan, she received first‐line carboplatin, etoposide, and durvalumab. She developed immune‐related hypothyroidism after the first cycle and started levothyroxine. Following three cycles, ProGRP increased from 2860 to 5940 pg/mL, and contrast‐enhanced CT demonstrated primary tumour enlargement and new liver metastases, meeting RECIST 1.1 criteria for progressive disease. Brain MRI at that time showed no metastases. She then received second‐line amrubicin monotherapy, achieving a partial response after two cycles and further tumour shrinkage by cycle six according to RECIST 1.1; however, treatment was discontinued the patient's request due to persistent Grade 2 dysgeusia despite zinc supplementation and otolaryngologic evaluation.

**FIGURE 1:**
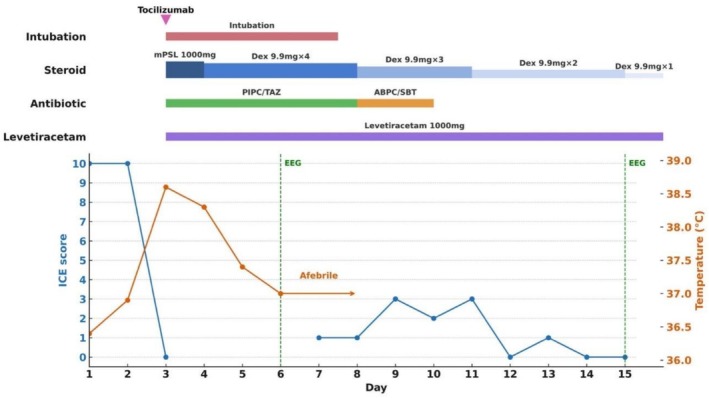
Clinical timeline. ABPC/SBT, ampicillin/sulbactam; DEX, dexamethasone; EEG, electroencephalogram; ICE score, immune effector cell‐associated encephalopathy score; mPSL, methylprednisolone; PIPC/TAZ, piperacillin/tazobactam.

**FIGURE 2:**
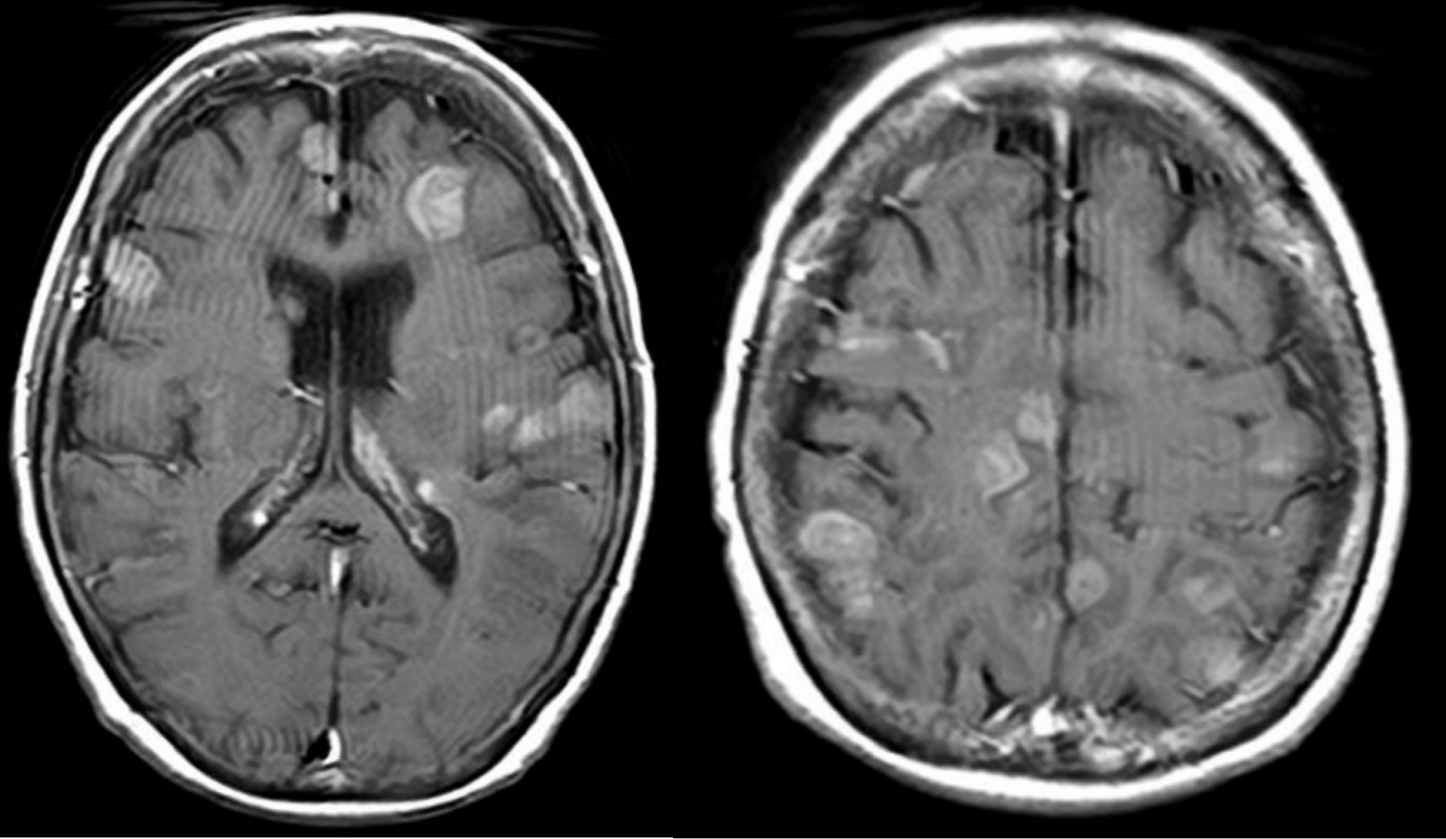
Contrast‐enhanced brain MRI on Day 7 post‐tarlatamab administration. Multiple relatively homogeneous enhancing masses were found in the brain parenchyma, consistent with multiple brain metastases.

On admission, her Eastern Cooperative Oncology Group performance status was 1, vital signs were normal, and her baseline immune effector cell‐associated encephalopathy (ICE) score was 10 [1]. Because tumour markers had been declining during second‐line therapy and she exhibited no neurological symptoms, brain MRI was not repeated immediately before initiating tarlatamab. The next day, tarlatamab 1 mg was administered. Fifteen minutes after infusion started, she developed a dry cough; the infusion rate was reduced and the dose was completed. Eighteen hours later, she developed fever (38.6°C) and her ICE score decreased from 10 to 0; she was responsive but aphonic. She then developed eyelid and left‐hand twitching consistent with focal seizures, which responded to intravenous diazepam (5 mg), although impaired consciousness persisted. High‐dose methylprednisolone (1000 mg) was given immediately. Approximately 1 h later, she developed generalized tonic–clonic seizures; a second dose of diazepam (5 mg) terminated the seizure, but her mental status did not improve. Grade 4 ICANS was diagnosed. Because of recurrent seizures, emergent tracheal intubation was performed for airway protection, and deep sedation was maintained for several days. Cytokine release syndrome remained Grade 1, but tocilizumab was administered due to concern for potential clinical deterioration. Levetiracetam (1000 mg/day) was started for seizure prophylaxis, and steroids were transitioned to intravenous dexamethasone 9.9 mg every 6 h. Head computed tomography showed faint high‐density lesions in both cerebral hemispheres suggestive of multiple brain metastases with minimal edema. She became afebrile on Day 4. Electroencephalography on Day 5 showed no epileptiform discharges. After adequate spontaneous breathing was confirmed, she was extubated on Day 6 (Figure 1). Contrast‐enhanced brain MRI on Day 7 revealed multiple enhancing parenchymal lesions (maximum diameter < 20 mm), consistent with multiple brain metastases (Figure 2). After extubation, her ICE score transiently improved to 3 but subsequently remained 0–1, and she was nearly non‐verbal. A repeat EEG on Day 14 showed no obvious abnormalities, and repeat head CT on Day 15 showed no reduction in brain metastases compared with Day 2. Her family declined whole‐brain radiotherapy, and best supportive care was chosen. Tarlatamab was not re‐administered because of the severity and prolonged course of ICANS. She had no history of allergies, asthma, drug reactions (including contrast media), or atopic disease, and no family history of allergic disorders; thus, no apparent allergic predisposition was identified as a contributing risk factor.

## 3 Discussion

Clinical trials of tarlatamab have reported ICANS as a notable toxicity that is typically reversible within days [2, 3]. In published studies, the incidence of ICANS appeared broadly comparable between patients with and without baseline brain metastases; however, a key limitation is that patients with brain metastases in pivotal trials had generally received prior brain radiotherapy. Real‐world series and case reports suggest that ICANS may be more frequent among patients with untreated brain metastases, although most reported events resolved relatively quickly [4, 5]. ICANS is thought to arise from excessive peripheral immune activation leading to endothelial injury and blood–brain barrier (BBB) dysfunction, which allows inflammatory mediators to enter the central nervous system and trigger neurotoxicity [1]. In patients with brain metastases, the BBB is often partially disrupted, making the metastatic microenvironment more susceptible to cytokine‐driven edema and inflammation during T‐cell activation induced by DLL3‐targeted therapy. Our case is distinctive for the combination of (1) brain metastases that were not recognized at the time of tarlatamab initiation and (2) life‐threatening, prolonged Grade 4 ICANS with persistent severely depressed ICE scores for weeks despite corticosteroids, antiseizure therapy, and intensive supportive care. Although the patient had undergone earlier brain imaging without metastases, subsequent MRI demonstrated multiple lesions after neurotoxicity emerged, raising concern that occult or newly developed intracranial disease may predispose to severe, prolonged ICANS. Neurotoxicity in this setting may also be challenging to interpret clinically, because brain metastases themselves can contribute to neurologic dysfunction; nonetheless, the abrupt onset within 18 h of tarlatamab and the accompanying seizure activity were consistent with ICANS. In addition, the patient had no allergic predisposition, no history of drug or contrast reactions, and no family history of atopic disease, suggesting that host allergic factors were unlikely contributors. The presence of untreated, structurally fragile metastatic lesions was therefore considered the most plausible risk factor for the unusually severe and prolonged ICANS observed.

This case highlights that untreated or unrecognized brain metastases may increase vulnerability to severe ICANS during tarlatamab therapy. Further real‐world data are needed to clarify risk factors and optimal management strategies for severe or prolonged ICANS.

## Author Contributions

**Hiroyuki Arai:** writing – original draft, visualization. **Shinji Sasada:** writing – review and editing, project administration. **Hiroki Kazahari:** investigation, writing – review and editing. **Yuya Homma:** investigation, writing – review and editing. **Kyohei Kaburaki:** writing – review and editing.

## Funding

The authors have nothing to report.

## Consent

The authors declare that written informed consent was obtained for the publication of this manuscript and accompanying images and attest that the form used to obtain consent from the patient complies with the Journal requirements as outlined in the author guidelines.

## Conflicts of Interest

The authors declare no conflicts of interest.

## Data Availability

The data that support the findings of this study are available from the corresponding author upon reasonable request.
